# Vientiane Multigenerational Birth Cohort Project in Lao People’s Democratic Republic: Protocol for Establishing a Longitudinal Multigenerational Birth Cohort to Promote Population Health

**DOI:** 10.2196/59545

**Published:** 2024-11-27

**Authors:** Jordyn T Wallenborn, Miley Sinantha-Hu, Vattahanaphone Ouipoulikoune, Sengchanh Kounnavong, Latsamy Siengsounthone, Nicole Probst-Hensch, Peter Odermatt, Somphou Sayasone, Günther Fink

**Affiliations:** 1 Department of Epidemiology and Public Health Swiss Tropical and Public Health Institute Allschwil Switzerland; 2 University of Basel Basel Switzerland; 3 Lao Tropical and Public Health Institute Vientiane Lao People's Democratic Republic

**Keywords:** Lao PDR, birth cohort, growth and development, mental health, dementia, exercise, behavior, aging, intergenerational, noncommunicable disease, hypertension, longitudinal cohort, low- and middle-income countries, maternal health, pregnancy, antenatal care, peripartum, postpartum, child health, infant health

## Abstract

**Background:**

Rapid global population growth and urbanization have led to an increase in urban populations in low- and middle-income countries. Although these urban areas have generally better health outcomes than lower-income rural areas, many environmental, social, and health challenges remain. Vientiane, the capital of Lao People’s Democratic Republic (Lao PDR), has approximately 1.5 of the 7.5 million Laotian population (2022) and provides a unique opportunity to examine health outcomes among socioeconomically diverse populations in the rapidly urbanizing context of the country.

**Objective:**

The aim of the Vientiane multigenerational birth cohort (VITERBI) project is to (1) establish a multigenerational birth cohort in Vientiane capital, Lao PDR, which is representative of the local population, (2) serve as the basis for additional observational (ie, cross-sectional) and intervention studies that promote population health in Vientiane province, and (3) investigate the social, epidemiological, and medical problems of public health importance to Lao PDR.

**Methods:**

VITERBI is a prospective multigenerational birth cohort. The study population is structured around children born between July 1, 2022, and June 30, 2023, who reside in Chanthabuly, Sikhottabong, Sangthong, or Mayparkngum districts of Vientiane. Whenever possible, children and their mothers are enrolled during pregnancy; nonreported pregnancies are enrolled after birth. The cohort plans to enroll 3000 pregnant women and their children and the infants’ fathers, grandparents, and great-grandparents for a total study population of approximately 13,000 individuals. Participants will be followed throughout the life course with a range of data collected, including demographics, behavior, diet, physical activity, physiology, neurodevelopment, health history, quality of life, environmental exposures, depression, anxiety, stress, resilience, household characteristics, obstetric history, birth outcomes, and various living and dementia scales for older adults. Biomarkers collected include height, weight, blood pressure, and hemoglobin levels. Currently, no statistical analyses are planned.

**Results:**

As of April 2024, this study has enrolled 3500 pregnant women and 4579 family members. Study participation is ongoing until May 2025 at minimum, with the goal to extend follow-up until 2050.

**Conclusions:**

The study cohort will be used as a basis for further observational (cross-sectional, longitudinal) and intervention studies. It also serves as a tool to investigate social, epidemiological, and medical problems of public health importance to Lao PDR, which will contribute to broader understanding of regional and international contexts.

**International Registered Report Identifier (IRRID):**

DERR1-10.2196/59545

## Introduction

The Lao People’s Democratic Republic (Lao PDR) is a socialist republic in southeast Asia, bordered by China, Vietnam, Cambodia, Thailand, and Myanmar. In 2018, Lao PDR was home to approximately 7 million people and at least 47 distinct ethnic groups [1]. Roughly half (52.5%) of the population identify as ethnic Lao [1], and one-third (35.6%) live in an urban area [2]. The majority of the urban population reside in the capital city of Vientiane [3]. Historically, Lao PDR’s economy has relied heavily on agriculture; more recently, tourism, foreign investments, and foreign aid have supported Lao PDR’s major economic growth—with an estimated 7% growth rate in gross domestic product between 2011 and 2017 [4]. In 2011, the World Bank changed Lao PDR’s economic status from a low income to lower middle-income economy [5].

Despite the rapid recent economic growth in Lao PDR, many environmental, social, and economic factors contribute to the high rates of mortality and morbidity [2]. Lao PDR reports a high incidence of infectious diseases, including malaria [6,7], dengue [8], Japanese encephalitis [9], typhoid [10], and tuberculosis [7,11]. Parasitic diseases endemic to Lao PDR, including schistosomiasis, soil-transmitted and food-borne helminthiases, and taeniasis, are classified by the World Health Organization (WHO) as neglected tropical diseases, and these disproportionately impact impoverished communities and women and children, engendering broader social and economic consequences [12]. Only 55% of the Laotian population has access to safe drinking water, which increases the frequency of preventable water-related diseases [13,14]. In recent decades of economic development, noncommunicable diseases have increased significantly, among which diabetes mellitus, cardiovascular disease, and various cancers are of particular concern in Lao PDR.

Maternal and child health is a strong predictor for population health and the development and sustainability of economies [15]. Lao PDR has the highest maternal mortality rate in Southeast Asia—estimated between 400 and 600 deaths for every 100,000 births from 2000 to 2008 [16]. Approximately 2 in 5 children younger than 5 years have moderate or severe stunting [17]. On average, a child of poor health will have a lower education attainment and earn a lower income, usually following a similar social and health trajectory as the mother [18]. Although the determinants of maternal and child health are vast and multifaceted, epidemiologic research utilizes several indicators to measure the maternal and child health status of a country, such as low birth weight, maternal morbidity, and antenatal or postpartum care attendance [19].

Maternal and child health indicators are useful tools for guiding public health strategies to improve population health; however, collecting data on maternal and child health alone is not sufficient to understand the health needs of populations. Intergenerational health and wealth beyond the mother also directly influence the life course trajectory of children [20-22]. Mental health problems in childhood are directly related to both the parents’ and grandparents’ mental health history and current mental health status [23]. Children born into socioeconomically disadvantaged families are more likely to have problems with language and reasoning, and similar challenges are found among family members within 2 generations of the family lineage [24]. Epigenetic modifications for various diseases, including gestational diabetes, are impacted by both family genetic backgrounds and environmental factors [25], highlighting the importance of combining intergenerational and individual maternal and child health data.

To date, only a few cohorts exist in Lao PDR or Vientiane. A prospective, community-based cohort study in Vientiane (N=4885 participants, 995 households, 25 villages) was conducted from 2015 to 2016 to monitor influenza-like illnesses and its causative pathogens [26]. Another prospective cohort study in Vientiane (n=1000 pregnant women) was conducted from 2013 to 2015 to investigate the association between maternal morbidity, small for gestational age, and fever [27]. To the best of our knowledge, there are currently no active cohort studies covering all age ranges in this setting.

Our Vientiane multigenerational birth cohort (VITERBI) is the first multigenerational birth cohort created in the urban and semiurban districts of Vientiane capital, Lao PDR, focused on intergenerational health and life course trajectories of children, their parents, grandparents, and great-grandparents. Cohort data will be analyzed to quantify key health challenges and gaps across population groups, ultimately to develop interventions and social impact programs for the improvement of population health.

VITERBI has 3 main aims:

1. Establish a multigenerational birth cohort in Vientiane capital, Lao PDR, which is representative of the local population.
2. Serve as the basis for additional observational (ie, cross-sectional) and intervention studies that promote population health in Vientiane province.
3. Investigate the social, epidemiological, and medical problems of public health importance to Lao PDR, including intergenerational prevalence of overnutrition and undernutrition, access to antenatal care, adult access to health care, prevalence and predictors of communicable and noncommunicable diseases within families, the current status of geriatric care, and its socioeconomic determinants.

## Methods

### Study Design

The VITERBI cohort will be established in the Vientiane capital of Lao PDR and is a prospective longitudinal birth cohort study enrolling children (during pregnancy), their parents, and relatives up to 3 generations (maternal and paternal grandparents and great-grandparents). Four districts of Vientiane capital were purposely selected based on overall socioeconomic status: Chanthabuly and Sikhottabong are 2 districts located in urban areas of high socioeconomic status, while Sangthong and Mayparkngum are 2 districts located in the semiurban, less developed areas of low socioeconomic status.

### Participants

The target sample size for the VITERBI cohort is 3000 children and their relatives (~13,000) across 4 selected districts. Index women (ie, pregnant women and the first participant enrolled within a family) are eligible for recruitment if they live in Vientiane capital in 1 of the 4 study districts; had an expected due date or gave birth between July 1, 2022, and June 30, 2023; do not plan to permanently move outside the study area; do not have a medical, intellectual, or psychological disability; and agree to participate with a signed informed consent. If the participant is younger than 18 years, a legal representative will additionally need to sign the informed consent. Whenever possible, women will be enrolled during pregnancy; however, women who were not enrolled during pregnancy will be enrolled shortly after birth by using a modified late enrollment questionnaire.

**Figure 1:**
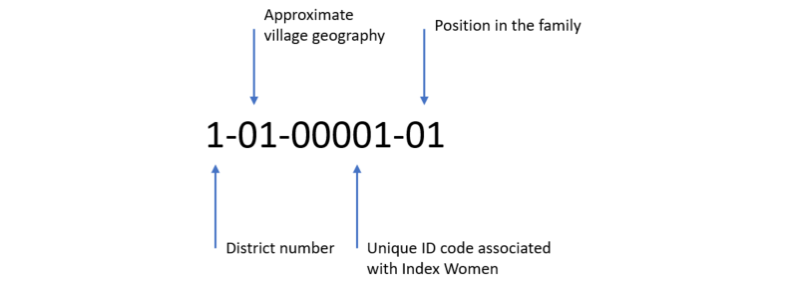
Participant ID interpretation of the Vientiane multigenerational birth cohort (VITERBI) participants.

Index women are tracked with a unique VITERBI ID number within the project database. All subsequent family ID numbers retain the number associated with the index woman in addition to their specific unique ID (Figure 1). Thus, all unique personal ID numbers assigned to family members of the child will be associated with the original ID number of the index woman. The ID numbers also indicate the district of residence of the index woman.

**Figure 2:**
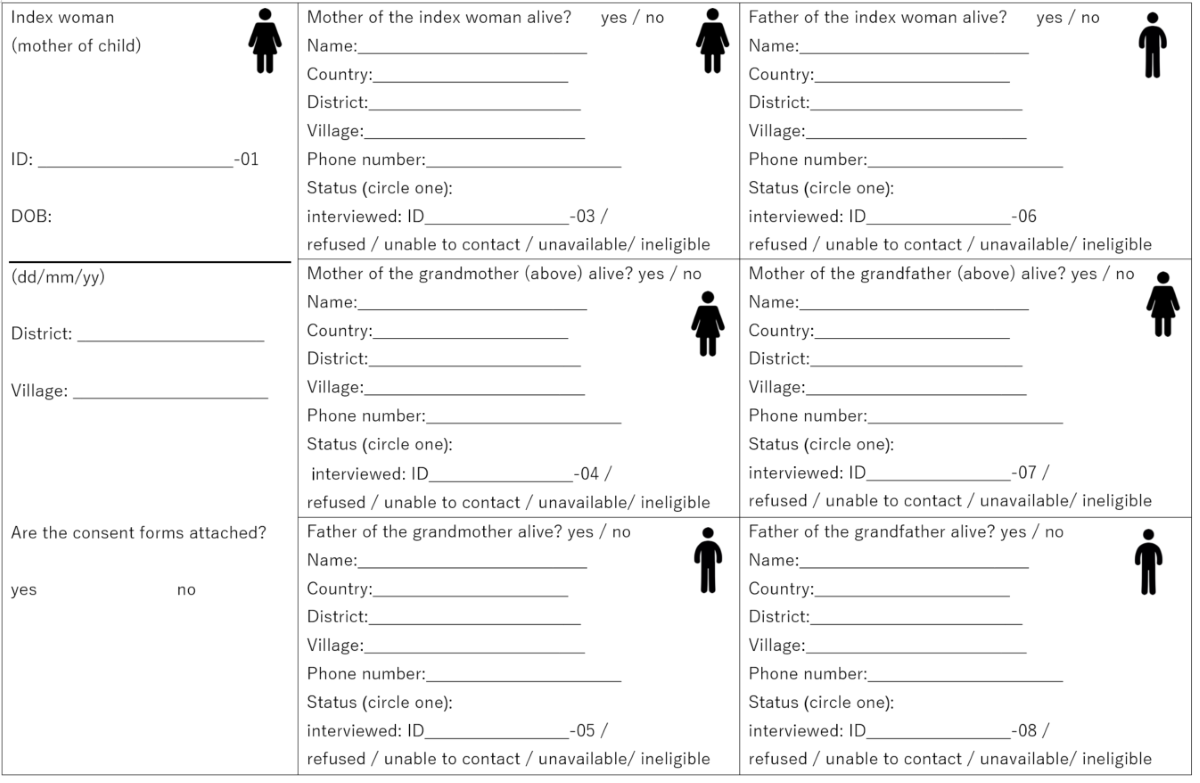
Family tree of the index women of the Vientiane multigenerational birth cohort (VITERBI). DOB: date of birth.

Enrollment data from each family member will be used to construct a family tree based around the index woman and child (Figure 2). All enrolled members will be followed up for a minimum of 1 year, with the ambition to extend the follow-up period until 2050. Members of the established cohort will also be recruited and enrolled into additional studies, as they arise.

### Ethics Approval

Ethics approval for this study was obtained from the Ethics Commission of Northwestern and Central Switzerland (EKNZ, 2020-00037) and the National Ethic Committee of Health Research, Ministry of Health, Lao PDR (035/NECHR). This research project will be conducted in accordance with the protocol, Declaration of Helsinki [3], Swiss Human Research Act, and Swiss Human Research Ordinance [1] as well as other locally relevant regulations.

### Informed Consent

Eligible participants will be visited initially by study staff. The purpose of this study and its procedures will be explained to participants, and a written informed consent will be obtained. Pregnant women will give consent for themselves and their newborns. For minors (age<18 years), both the pregnant woman and her parents will provide consent. For participants who are illiterate, there will be an additional witness who is not part of the study team. Participation is voluntary, and patients have the right to withdraw from the study at any given point in time with no further obligations. If the participant withdraws at any time, their data will be deleted. Confidentiality of information will be assured to the participants. All participants younger than 18 years are considered vulnerable populations according to the Lao PDR legislation. If the respondent is underage and agrees to participate, he/she needs the authorization of a parent or legal guardian who cosigns the study informed consent.

### Recruitment

We used a variety of sampling methodologies to identify all births between July 1, 2022, and June 30, 2023. To start, we embedded VITERBI recruitment strategies within the existing Laotian health care system. In Lao PDR, public health care services are delivered according to administrative levels, that is, central/national, provincial, district, and village, which operate under the centralized Ministry of Health. We recruited eligible pregnant women from hospitals operating at all 3 levels of care available in Vientiane capital: central/national, district, and village. Health care staff at public health care facilities at the central and district levels facilitate recruitment by notifying VITERBI staff of eligible participants and connecting consenting individuals with VITERBI staff.

VITERBI staff are assigned in teams to 1 of the 4 study districts to conduct recruitment. VITERBI staff partner with local staff working in public health and governance at both the district and village levels through institutional relations formalized by the Lao Tropical and Public Health Institute, a Lao governmental research entity administered by the Lao Ministry of Health. These partnerships involve working closely with key informants such as hospital leadership, health care providers, public administrators, village health volunteers, and local leaders of all administrative levels. Local collaborators generally keep more granular records (eg, unofficial village census) than national partners and maintain situational awareness of their populations’ health status. Key informants from each village within the 4 study districts work alongside VITERBI staff to identify and recruit potential participants. VITERBI staff regularly visit antenatal care clinics known to serve populations that reside in the study districts to recruit eligible women. Enrollment and all data collecting activities of the VITERBI cohort study are coordinated from a central office at the Lao Tropical and Public Health Institute.

**Figure 3:**
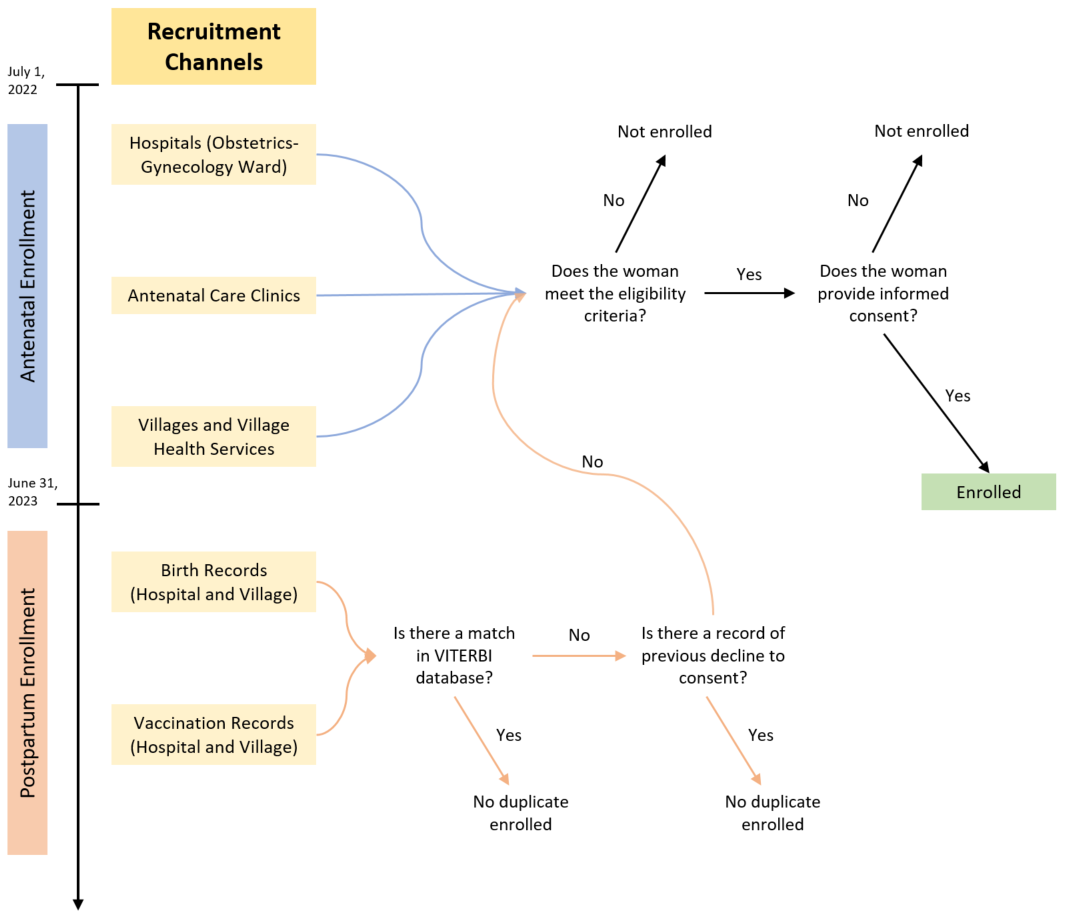
Enrollment pathway of the Vientiane multigenerational birth cohort. VITERBI: Vientiane multigenerational birth cohort.

Women who were not enrolled during pregnancy are enrolled postpartum to increase enrollment coverage. Birth records are obtained from central hospitals and local health centers to identify recent births that occurred within the cohort window. Staff attempt to contact these women who were neither enrolled nor declined a previous recruitment attempt during pregnancy. Study staff also obtain vaccination records from central health entities to cross-check infant enrollment with both on-time and postpartum recruitment and attempts to recruit women who do not appear in either channel (Figure 3). Access to these records is facilitated by the Lao Tropical and Public Health Institute through the Ministry of Health. Formal birth records obtained from hospitals and vaccination records from the National Immunization Program are used to evaluate recruitment rates. Increasing the overall enrollment coverage of eligible women (prepartum and postpartum) assures that the cohort represents the target population.

Index woman enrollment is the cornerstone for subsequent enrollment of the child’s relatives. In the field, VITERBI staff work closely with the index women to construct family trees detailing relatives of the child who are alive, eligible (living in the 4 study districts), and available to take part in the study. The family tree document guides staff recruitment efforts by providing basic demographic (name, age, and relationship to the child) and contact information of all family members who can be included. As we recruit additional family members, we anticipate their input will continue to guide recruitment and interview of subsequent members by reviewing the family tree document.

### Data Collection Instruments

The VITERBI study has a total of 6 questionnaires: (1) child enrolled during pregnancy (ie, index women), (2) child enrolled after birth (late enrollment), (3) postpartum form to collect birth outcomes, (4) adult questionnaire for the child’s father, grandparents, and great-grandparents, (5) a short questionnaire for family members who are incapacitated or have a cognitive impairment, and (6) household characteristics. Table 1 shows extensive information about the data collection instruments. All surveys are administered in-person via tablets by trained staff in the field and uploaded remotely to a web-based server at the end of the workday. Each questionnaire is briefly described below but can be accessed by reasonable request.

**Table 1:** Data collection for each participant in the Vientiane multigenerational birth cohort (VITERBI).

Question type			Participants										
			Index mother		Late enrollment		Index mother postpartum		Adult^a^ (<60 years)		Adult^a^ (≥60 years) with no cognition problem		Adult^a^ (≥60 years) with cognition problem
Demographics			✓		✓				✓		✓		✓
Employment			✓		✓				✓		✓		
Obstetric history			✓		✓				✓^b^		✓^b^		
Antenatal care			✓		✓								
Prenatal supplementation			✓		✓								
Tobacco use			✓						✓		✓		
Alcohol consumption			✓						✓		✓		
General diet			✓						✓		✓		
Dietary salt			✓						✓		✓		
Handwashing practices			✓						✓		✓		
Raw food consumption			✓						✓		✓		
Physical activity			✓						✓		✓		
Transportation			✓						✓		✓		
Sedentary behavior			✓						✓		✓		
Pattern recognition and digit span			✓						✓		✓		
Disability			✓						✓		✓		
History of raised blood pressure			✓						✓		✓		
History of diabetes			✓						✓		✓		
History of raised cholesterol			✓						✓		✓		
History of cardiovascular disease			✓						✓		✓		
Experience of pain and arthritis			✓						✓		✓		
Chest pain			✓						✓		✓		
Cervical cancer screening			✓						✓^b^		✓^b^		
Health history			✓						✓		✓		
Environmental exposure			✓						✓		✓		
Family survival history			✓		✓				✓		✓		
Quality of life			✓						✓		✓		
Depression, anxiety, stress			✓						✓		✓		
Resilience			✓						✓		✓		
Recent birth outcomes					✓		✓						
Breastfeeding initiation, exclusivity, duration					✓		✓						
Postpartum depression					✓		✓						
Postpartum medical visit					✓		✓						
Instrumental Activities of Daily Living Scale											✓		✓
Activities of Daily Living													✓
Revised Hasegawa Dementia Scale											✓		✓
**Physical measurements**													
	Blood pressure	✓		✓		✓		✓		✓			
	Heart rate	✓		✓		✓		✓		✓			
	Weight	✓		✓		✓		✓		✓			
	Height	✓		✓		✓		✓		✓			
	Waist circumference	✓		✓		✓		✓		✓			
	Hip circumference	✓		✓		✓		✓		✓			
	Leg length	✓		✓		✓		✓		✓			
	Trunk length	✓		✓		✓		✓		✓			
	Mid-upper arm circumference	✓		✓		✓		✓		✓			
	Hemoglobin	✓		✓		✓		✓		✓			

^a^Relation to the child: father, grandmother, grandfather, great-grandmother, and great-grandfather.

^b^Women only.

### Index Women

Women enrolled during pregnancy were asked about their pre–pregnancy weight in kilograms, antenatal care, obstetric history, and plans for infant feeding. If enrollment occurred after birth, a shortened questionnaire was provided so as not to interfere with the mother-infant bonding (late enrollment questionnaire).

### Postpartum Interview

The postpartum questionnaire obtains information about the child’s birth outcome, health status, and general obstetric and postpartum experiences. Breastfeeding initiation and duration are assessed using questions from the Infant Feeding Practices Survey II [28]. Postpartum depression is assessed using the Edinburgh Postnatal Depression Scale [29]. Only when the women moved to a district outside the VITERBI catchment area were the questionnaires completed via telephone.

### All Adult Family Members

Utilizing validated questionnaires, we compiled an extensive interview to assess population characteristics. Each questionnaire includes the following information: demographic information; behavioral measurements for tobacco use, alcohol consumption, diet, and physical activity; health history; physical measurements, including blood pressure, height, weight, and waist circumference; sections from the WHO stepwise approach to surveillance [30]; hemoglobin levels by using the HemoCue Hb 301; family survival history; self-rated general quality of life [31]; environmental exposures to pesticides and cooking conditions; general mental health assessed using the Depression Anxiety Stress Scales [32,33]; cognitive status using progressive matrices [34] and the digit span [35]; and resilience levels [36]. Only women are asked the cervical cancer screening question from WHO stepwise approach to surveillance [30].

### Older Adults (Aged 60 Years or Older)

Individuals aged 60 years or older complete additional aging-related modules: Activities of Daily Living (ADL) and Instrumental Activities of Daily Living Scale are assessed using the Barthel [37] and Lawton [38] index, respectively. The Revised Hasegawa Dementia Scale [39] is used to assess cognitive impairment. If there is any indication that the adult is cognitively unable to complete the full-length interview, a short questionnaire only including these indicators was included. The main caretaker would answer questions regarding ADL. Only ADL was assessed among adults who had a main caretaker.

### Household Questionnaire

The Demographic and Health Surveys Phase 8 Questionnaire for household characteristics is used to assess access to drinking water, toilet facilities, mosquito net use, wealth and assets, food security, and use of social services.

### Retention of Participants

We collect a variety of contact information to reduce participant attrition. Participants provide their first and family names, address, phone number, email address, village leader name, temple or religious organization, and the contact information of a family member or close friend. In addition, we provide a small gift for participation. Immediately after the physical examination during baseline interview, all participants receive their biometric test results. Results include blood pressure, hemoglobin levels, and waist and hip circumference values. Results are explained during the session by licensed medical personnel (eg, staff nurses) and written on an information pamphlet for future reference. If the test results are abnormal, participants will be referred to their local health center for treatment. In addition, all participants who complete the baseline questionnaire receive a small gift (ie, hand soap) that equates to approximately US $1.

### Data Management

All questionnaires and data management are conducted in Open Data Kit (ODK). ODK is an open-source Android app that supports mobile data collection services. ODK Collect replaces paper forms used in survey-based data gathering and can be utilized without network connectivity. It supports a wide range of question types and answer formats. All data collection will occur offline on password-protected Android devices. Data connectivity will only be used to send data collected in the field at the end of the workday, when the data collection staff are in network range. Data storage is on a local secured server at Swiss Tropical and Public Health Institute by using the ODK Aggregate server.

### Statistical Analyses

The main goal of our study is to provide descriptive epidemiologic information on the current health status of a representative, purposive sample of children and their families in Vientiane province, Lao PDR. This includes incidence and prevalence estimates, geospatial maps displaying disease distributions, and factors of health and health behaviors (ie, accessing health care). Currently, no statistical analyses are planned. However, any additional research study will submit a separate institutional review board application.

## Results

As of April 2024, this study has enrolled 3500 pregnant women and 4579 family members. Study participation is ongoing until May 2025 at minimum, with the goal to extend follow-up until 2050. The cohort has already served as a basis for additional observational and intervention studies that promote population health in Vientiane province: (1) VITERBI GUT, a prospective birth cohort studying the link between early life malnutrition, the microbiota, and metabolic health [5] and (2) a randomized controlled trial providing social transfers to increase exclusive breastfeeding and promote maternal and child health [40].

## Discussion

Multigenerational prebirth cohorts provide in-depth information about pregnancy, infants, early childhood, intergenerational health and wealth cycles, and etiology for specific diseases. Conducting longitudinal cohort studies in low-and middle-income countries provides unique challenges that are often not seen in high-income nations.

One key challenge in the specific context of Vientiane capital is the lack of robust information technology systems that would facilitate official data sharing and provide patients with personal records. Although decentralized operations might improve health system equity in hard-to-reach places subject to remote conditions and limited infrastructure, widespread deficiency of interfacility and interagency communication also results in fragmentation of the health system [41]. The VITERBI recruitment methodology employs diverse strategies in an attempt to reach eligible women and their families across different channels of the public health care and municipal systems. Of note, the recruitment efforts of women seeking and receiving care at 2 national facilities, Mother and Child Hospital and the maternity ward of Mahosot Hospital, yielded higher enrollment compared to province-level and district-level facilities. Face-to-face contact with local health staff and officials is an effective strategy for identifying eligible participants, as centralized data sharing and communication are not effective strategies in Lao PDR. Although record-keeping may vary between villages and districts, local staff generally have knowledge and records—both formal and informal—of pregnancies within their jurisdiction. Basing their work out of one of the study districts for prolonged periods, VITERBI staff are able to develop consistent partnerships with local staff at both the district and village levels. This strategy is particularly useful in recruiting women who may not be accessing health services within the previously described VITERBI contact circuit (eg, who are accessing private services or are not accessing any health care services).

Despite the varied and often fragmented channels for identifying and recruiting eligible participants, close collaboration with stakeholders at all levels, detailed study coordination from our team, and adaptive communication and working strategies have helped increased enrollment. Although there likely remains untapped avenues for reaching and recruiting eligible women, the previously described methods currently push the operational limits of our administrative and fieldwork staff. These staff members contend with the need for persistent communication with local health authorities, receive redundant and disorganized population data, and work around convoluted timelines set by administrative bottlenecks. These challenges emphasize the need to develop adaptable workflows with flexible coordination and strong collaboration among the staff.

Further challenges to producing a clean and robust database for a cohort of this magnitude include maintaining record completeness and reconciliation between the web-based questionnaire database and physical forms. When physical forms are collected (eg, participant consent forms, family trees) from the field, they are submitted biweekly to the main office to be double-checked and filed. This check is essential to ensure that no duplicate ID numbers have been assigned to different index women—an error that occurs due to misprinting of questionnaire codes associated with the IDs and the dissemination of misprinted forms to interview staff working in different groups in different areas of the same district. Duplicate ID numbers are detected by thorough check of the physical forms and then amended in the web-based database by manually assigning a new unique VITERBI ID in ODK.

All forms, including physical consent and contact forms alongside digital survey forms, must be checked for completeness. Then, physical and web-based forms must be compared with one another to ensure essential information (name, date of birth, contact phone number, district of residence, etc) match to the best of our ability. The Lao language and naming system presents unique challenges, as there is no standard Romanization, and often an individual is identified by an informal name that does not match legal or medical records. These factors result in multiple spellings of the same name in English translation and several names being associated with one individual. Often, informal names are quite generic, which can lead to confusion if there are no additional formal names or surnames listed for the participant. For this study, we collate all the records and associate as much identification information as is available with the unique VITERBI ID numbers. Thus, we rely on the index numbers to identify participants.

Many lessons learned from VITERBI can be used for other cohort studies in low-resource settings. These lessons include:

1. Local capacity building and continuous strengthening is essential. A thorough training of all research staff is critical for minimizing data collection errors. Creating sustainable institutional partnership for comparative cohorts of mutual benefit for different partners is also key for future research.
2. Building trust with local leaders facilitates identification of participants and creates community ownership of the cohort goals.
3. In minority populations such as the Hmong ethnic group in Vientiane, utilizing local staff that reflect the community population who act with high cultural competency from villages or neighborhoods is important for engaging difficult-to-reach populations.
4. Flexibility and creativity in research staff are key. Participant identification is difficult, and the local study staff may need to adapt recruitment strategies in order to engage the population. Many of the villagers were only available in the evenings or on weekends, creating the need for staff to stay for long periods of time within the community.
5. Study instruments need to be reflected across many different age and ethnic groups. The multigenerational aspect of the cohort calls for a multidisciplinary team to ensure cohesion across study questions.
6. The medical system is overburdened, and medical records are not digitized. Collecting handwritten ledgers to identify and track children and their mothers is time-intensive and requires a formal data tracking system.

Each community offers unique challenges. In low-resource settings where longitudinal studies are more difficult, such data would often be the most beneficial for population health. For this exact reason, additional longitudinal studies should be conducted in these settings. Although the researcher should not be complacent in recognizing the specificity of the study setting, the methods and lessons described here may prove relevant in the formation of future studies in the Lao PDR context and beyond.

## Abbreviations

ADL: Activities of Daily Living
Lao PDR: Lao People’s Democratic Republic
ODK: Open Data Kit
VITERBI: Vientiane multigenerational birth cohort
WHO: World Health Organization

## Data Availability

Data sharing is not applicable to this paper, as no datasets were generated or analyzed during this study.
